# The effect of *Camellia Sinensis* (green tea) mouthwash on plaque-induced gingivitis: a single-blinded randomized controlled clinical trial

**DOI:** 10.1186/2008-2231-20-39

**Published:** 2012-09-24

**Authors:** Niloofar Jenabian, Ali Akbar Moghadamnia, Elaheh Karami, Poorsattar Bejeh Mir A

**Affiliations:** 1 Department of Periodontology & Implantology, Dental Materials Research Center, Dentistry School, Babol University of Medical Sciences and Health Services, Babol, Iran; 2 Department of Pharmacology, School of Medicine, Babol University of Medical Sciences and Health Services, Babol, Iran; 3 General Dentist, Private Practice, Babol, Iran; 4 Dentistry School, Dentistry Student Research committee(DSRC), Dental Materials Research Center, Babol University of Medical Sciences and Health Services, Babol, Iran

**Keywords:** Camellia Sinensis, Gingivitis, Complementary medicine, Clinical trial, Mouth wash

## Abstract

**Abstract:**

Background and the purpose of the Study

Complementary medicine received high attention during last decades. We aimed to assess the efficacy of Green tea mouthwash on plaque-induced gingivitis as the most common form of periodontal disease.

**Methods and materials:**

We designed a single blinded placebo controlled clinical trial. High school female students with chronic generalized plaque-induced gingivitis were distributed to receive either 5 ml of Green tea 5% two times/day or normal saline with the same dosage. Gingival index (Sillness & Loe), plaque index (Sillness & Loe) and bleeding index (Barnett) were recorded at baseline and five consecutive weeks. Comparisons were made by a general linear model, repeated measure ANOVA and a Bonferroni test applied for multiple comparisons.

**Results:**

Twenty five students were recruited in each arm of the study. A significant improvement was observed in all periodontal indices during the study (P < 0.001). Two groups were contrasted by changing patterns of alteration of indices (P < 0.05). Although total amount of improvement was higher in mouthwash group, the differences did not reach a statistically significant level (P > 0.05, observed power for GI: 0.09, PI: 0.11 and BI: 0.07).

**Conclusion:**

Green tea mouthwash may be a safe and feasible adjunct treatment for inflammatory periodontal diseases. A future larger scale study is warranted for better evaluating the effect of green tea.

## Introduction

Gingivitis and periodontitis, as the multifactorial diseases, are mainly derived by interaction between invasions of causative bacteria and host immune response of varied degrees [1]. Plaque induced gingivitis is the most common form of gingivitis and is induced by accumulation of microbial plaque containing more than 300 types of bacterial species [2]. Historical periodontal treatment dates back to 30_B.C,_ when vinegar was applied to cure periodontal diseases. Over the time, more specific treatment modalities were developed, including scaling and root planning (SRP) and surgical techniques [2].

Complementary medicine received a great attention during recent decades which recommends supplementation with various ingredients. Diverse modes of delivery including chewing candy, chewing gum, dentifrices and local drug delivery strips are introduced [3-6]. Tea and in particular, green tea are among most popular beverages, with high daily consumption in Asia and especially in Iran. Several properties including antioxidant, anticaries, antibacterial, antiviral, antidiabetic, antimutagenic and antitumural properties are addressed for green tea [3]. Green tea, *Camellia Sinensis* from the family of *Thea Cease*[7] is mostly cultivated in coasts of Caspian sea in North of Iran. Its remedial effects are associated with the polyphenol contents comprising catechin (C), epicatechin (EC), gallocatechin (GC), epigallocatechin (EGC), epicatechin gallate (ECG), and epigallocatechin gallate (EGCG). The two latter are mainly found in green tea rather than the black tea and are among most potential contents to be reviewed for periodontal adjunct therapies in terms of their special anti-collagenase activity [8,9]. In addition, it is suggested that EGCG inhibits the growth and cellular adherence of periodontal pathogens [10].

Sole treatment with SRP may lack achievement in complete irradiation of red complex bacteria in depth of deep narrow pockets; hence adjunct chemical elimination for plaque induced periodontal diseases seems necessary. We aimed to investigate the effect of green tea mouthwash in plaque-induced gingivitis, the most common form of plaque-related periodontal diseases.

## Methods

### Enrollment

A Single-blinded (i.e., assessor and analyst) randomized placebo-controlled clinical trial was conducted among female high school students aged 14-16years in Babol discrete during 2009. Students with chronic generalized plaque-induced gingivitis were enrolled. Those having systemic illness, antibiotic consumption during last three month, known allergy to tea derivatives and concurrent medications with known effect on the periodontium (e.g., oral contraceptive, antibiotics, herbal medications, etc.) were excluded. This project was approved by the ethics committee of the Babol University of Medical Science (Protocol No.4678, 22.7.85). Subjects were provided with written informed consent and all researchers undertook Helsinki treaty.

### Interventions

Final recruited patients were distributed into two arms of the study; case patient that received green tea extract mouthwash 5% and the control group who received normal saline as the placebo. All patients were instructed to take the medication two times a day, each time 5 ml rinsed for 30 seconds. They were also taught to floss and also to brush their teeth three times daily with the Bass method (i.e., a brushing method including gingival sulcus entrance cleansing).

### Measures and End-points

All patients were examined and the plaque index (Loe & Sillness) [11], gingival index (Sillness & loe) [12] and bleeding index (Barnett) were recorded at the baseline and five consecutive weeks (i.e., total duration of study was five weeks). Primary endpoint was defined as the reduced gingival index at the end of clinical trial and secondary outcomes were intended as the improvement in bleeding and plaque scores. Moreover, patients were asked to report any side effect and were checked for intra-oral adverse effects in each appointment.

### Sample preparation

Green tea mouthwash was extracted from the plant *Camellia Sinensis* (Voucher No: 30_s/00280) in the laboratory of pharmacology department of Babol University of Medical Sciences by an expert pharmacologist. The physical and botanical characteristics of *Camellia Sinensis* have been confirmed by an expert pharmacologist and one small sample of the material was deposited in the laboratory for possible need in the future. Leaves of the plant were chopped, fragmented, and broken into small pieces, and each 100 g of leaves were soaked in 500 ml of methanol for 48 hours. Thereafter, the solution was passed through a strainer and was transferred to a plate. Plates were maintained in normal temperature of laboratory for 3-4 days, and then the crystal powder of extract was scraped from the plates. Finally green tea mouthwash 5% was prepared (0.5 g of extract in 100 ml distilled water) and poured into bottles each contains 240 ml. Normal saline mouthwash was prepared in the bottles with the same shape, capacity and color. Actually, preparing an inert placebo with the exact same color, odor and taste as green tea was not feasible. In addition, Normal Saline has a very limited effect on gingivitis and it was used only because of its rinsing effect (both group were the same in rinsing a mouthwash).

### Statistics

We estimated that 25 patients would be required to achieve 80% power to detect a standard difference of 1.5 between two groups in the rate of primary end point improvement (using Altman’s nomogram). Continuous data were expressed as mean. To trace the changing trends of periodontal indices, a general linear model (GLM) repeated measure ANOVA was built. Sphericity (one of ANOVA presumption) was tested with a Mauchly’test. In the case of violation, data were adjusted with an Epsilon Greenhouse-Geisser statistic. Moreover, in order to more precise analysis of paired groups, the post hoc multiple comparison was accomplished and a Bonferroni test was utilized.

## Results

**Figure 1:**
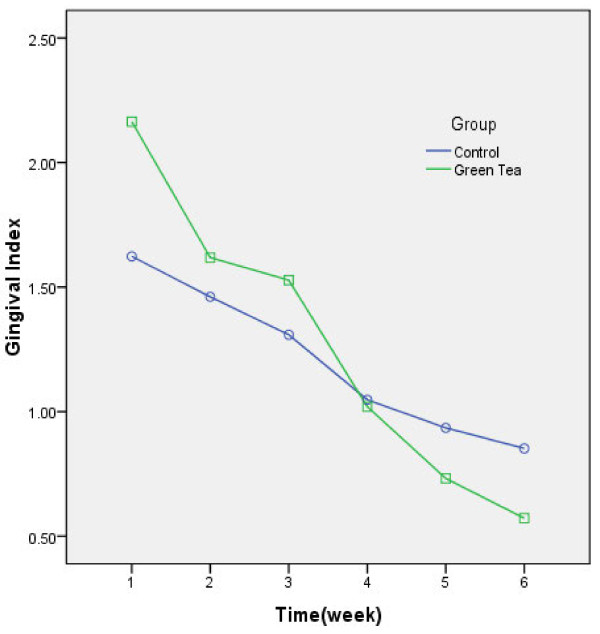
** Changing trends of Gingival index during the study. Week-to-week comparison revealed significant improvement except for 2**^**nd**^**to 3**^**rd**^**week (P = 0.27).**

**Figure 2:**
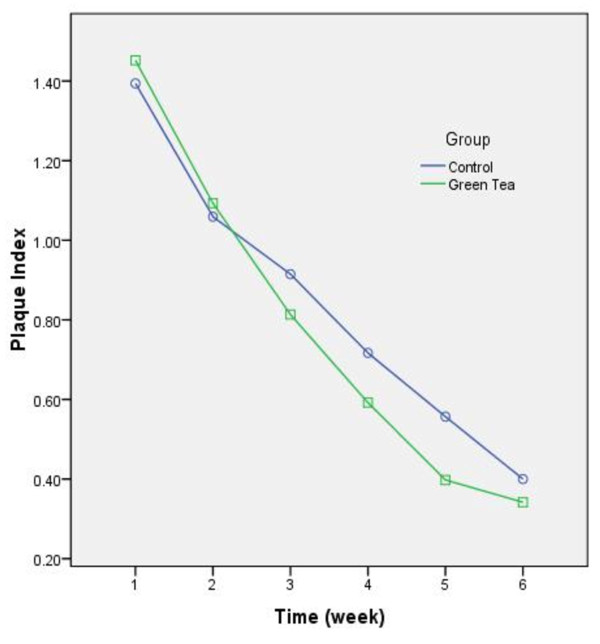
Changing trends of plaque index during the study. The index was improved significantly from each week to the following week (P < 0.001).

**Figure 3:**
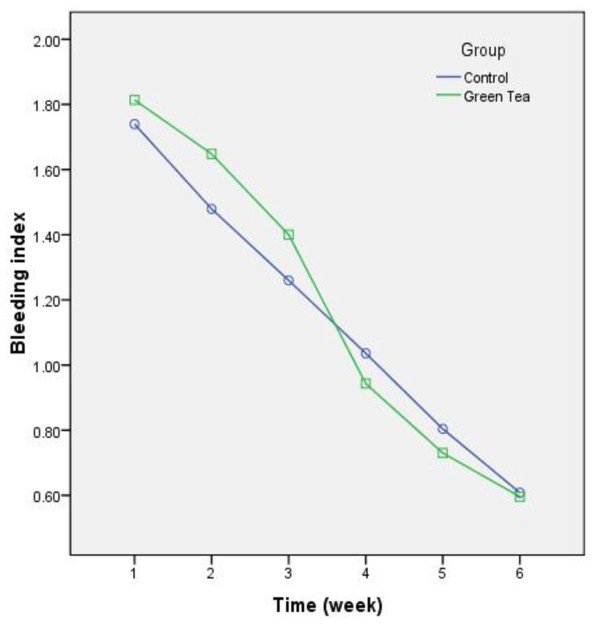
Changing trends of bleeding index during the study. The index was improved significantly from each week to the following week (P < 0.001.)

A total of 50 female high school students were recruited and distributed into case and control groups each consisting 25 patients. Detailed data regarding alteration of periodontal indices during the study are displayed in Figures 1,2,3.

### Primary endpoint

According to the recorded gingival indices, a significant improvement was observed in both groups (F(2.52) = 166.82, P < 0.001, observered power = 1). Overall changing trend in GI was different between the study groups (F(2.52) = 18.44,P < 0.001, observed power = 1), yet the mean difference from baseline to the 6^th^ week was not statistically significant (mean difference = 0.07, P = 0.54, observed power = 0.09). The week-to-week comparison revealed a remarkable decrease for all the comparisons (P < 0.001) except for differences between 2^nd^ and 3^rd^ week (P = 0.27).

### Secondary endpoints

Analysis clarified a significant decrease in BI and PI of both groups’ patient (PI: F(2.5) = 221.67, P < 0.001, observered power = 1, BI:F(3.48) = 373.03, P < 0.001, observered power = 1) and a contrasting pattern of recovery was observed between the study groups (PI: F(2.5) = 2.74, P = 0.02, observed power = 0.82, BI: F(3.48) = 5.33,P = 0.001, observed power = 0.98). Nevertheless the total baseline-to-6^th^ week difference was not significant (PI: mean difference = 0.06, P = 0.46, observed power = 0.11, BI: mean difference = 0.03, P = 0.63, observed power = 0.07). All inter-week comparisons were exceptional (P < 0.001). Despite of unpleasant taste, no adverse effects were reported during the study.

## Discussion

The present research investigated the effect of mouth wash containing green tea 5% on chronic generalized plaque-induced gingivitis. Our findings support the beneficial effect of green tea to improve inflammatory periodontal indices after five weeks of treatment. Furthermore, improvement of indices in control group is because of positive effects of saline in increasing oral hygiene. Recently, Kuvda et al. (2011) investigated the adjunct effect of SRP and locally delivered catechin via inserted strips into the surrounding pocket for a period of 21 days [3]. They reported a significant decrease in pocket probing depth (P < 0.001), yet PI and GI decreased insignificantly when compared to the SRP group (P < 0.05). In addition, a considerable reduction of causative bacteria was observed. This may point that those visible periodontal indices improvements are slightly behind elimination of bacterial. In our study, intercamparisons of GI revealed no differences (power = 0.09). In commitment with Kuvda et al., remarkable changes did not occur during the first 3 weeks. Lesser effect size of our investigation when compared to those of Kuvda et al. and Hirsawa et al. (2002) may be sought in different modes of medication delivery [3,10]. The continuous flow of gingival crevicular fluid and saliva wash out indeed decreases the efficacy of mouthwashes. In the contrary, Kuvda and Hirsawa applied a direct delivery system by means of implantation of drug strips into the depth of the pocket.

Improvements in plaque and bleeding indices applying green tea mouth wash are in commitment with many previous researches [4,13-17]. Intercamparisons for BI and PI did not reach a statistically significant level, yet changing trends were different between the study arms. Low achieved powers (PI: 0.11, BI: 07) indicate that a larger sample size may magnify such small differences. Moreover, methanol was used during drug preparation, however the final prepared medication was dried and a water based solution was prescribed. Consequently, the proportion of methanol is low with no considerable effect on periodontium and with probable anti-plaque effect preventing adhesion to tooth’ surface.

*Porphyromonas Gingivalis*, similar to other causative periopathogens, is proved to play a key role initiating the periodontal destruction. This microbe exerts its destructive characteristics by means of various protease including amionopeptidase, gingipains and collagenase (i.e., most potent) [18]. Indeed, oxidative-antioxidative capacity imbalance in favor of excessive oxidative stresses and inflammatory mediators would lead to advanced periodontal impairment [19]. Catechin contents specially, ECG, EGCG within green tea inhibits peptidase and collagenase activity [9,10,18]. In addition, they may subside proinflammatory cytokines such as interleukin (Il-1β) and tumor necrotic factor (TNFα) [20]. Moreover, by lowering Il-6 and decreasing expression of Cyr61, it inhibits bone resroption and thus osteolysis are observed [8,21]. Otake et al. suggested that green tea has an antiplaque effect due to the polyphenolic compositions and tannin compounds [22]. A small amount of tannin and vitamin K within green tea may improve bleeding index during the study [23]. Kushiyama et al. concluded that additive drinking of each cup of green tea, would decrease 0.023 mm of pocket depth, 0.028 mm of clinical attachment loss and would improve bleeding upon probing by 63% [24].

Our study is limited in its underpowered comparisons. Probably a better and precise comparison may be obtained from a larger scale study. In addition, the present research lacks the comparison between the effects of green tea mouth wash on various periodontal inflammatory indices with scaling and roots planing (SRP), as the gold standard of phase I of periodontal therapy. Indeed, subgingival irrigation using a sterile syringe would improve the efficacy of this medication with deeper intentional penetration to the depth of periodontal pockets [25]. The main goal of the present research was to test the efficacy of sole treatment with green tea, as a convenient and self-applicable means, in addition to reminding the proper method of brushing and flossing. It seems that such reminding and motivation served positively in treating gingivitis. In comparison to normal saline, green tea showed better effects, however this was not statistically significant. This may be discussed as the lacks of sensitivity of periodontal indices to detect milder degree of periodontium inflammation at the final weeks of intervention, compared to more sensitive and specific biomarkers (e.g., interleukins). Concurrent assessment of microbiologic and inflammatory biomarker would also better reveal the differences between interventional and control groups that are suggested for future researches.

## Conclusion

This study supports that the daily consumption of green tea mouthwash may be beneficial to cure or prevent gingival inflammation. Its prescription may be beneficial and of value in certain community groups such as adolescent students who are more affected by periodontal inflammation. It should be avoided in patients on anticoagulants and with advanced renal failure due to its vitamin K and aluminum content, respectively.

## Author’s contribution

Jenabian and Moghadamnia designed the study. Karami conducted the study. Poorsattar Bejeh Mir analyzed the data and contributed with Jenabian in literature search. All authors have participated in drafting, critical evaluation and approval of the final version.
